# Resilience and (Dis)empowerment: Use of Social Media Among Female Mainland Low-Skilled Workers in Macao During the COVID-19 Pandemic

**DOI:** 10.1177/21582440231160480

**Published:** 2023-03-15

**Authors:** Bei Ju, Hai Min Dai, Todd L. Sandel

**Affiliations:** 1 Macau University of Science and Technology, Taipa, Macao, SAR, China; 2 Shanghai Jiao Tong University, China; 3 University of Macau, Taipa, SAR, China

**Keywords:** resilience, (dis)empowerment, social media, female low-skilled migrant workers, COVID-19 pandemic

## Abstract

The role of social media in a resilient process is associated with the co-constitution of structural forces and users’ agency. During COVID-19, how women—particularly low-skilled labor migrants—used social media for empowerment is underexplored. By taking a socio-techno approach, this study qualitatively examines mobile phone-based social media usage among female mainland low-skilled workers in Macao when coping with the pandemic. The enabling yet constraining role of social media has been identified through semi-structured interviews. Social media use is a double-edged sword: on the one hand, social media is appropriated to relieve stress and anxiety, open access to updated COVID-19 related information, and manage contagious risks; on the other hand, it reinforces existing constraints and thus hinders resilience, due to female migrant workers’ high risk of addictive social media use and limited information literacy. Moving beyond the Information and Communication Technology empowerment, a more inclusive approach is recommended in the long term to cope with the risks and uncertainties posed by the pandemic.

On August 3, 2021, Macao’s 491-day record of having no COVID-19 infections ended when four locals from the same family tested positive. From 3:30 pm that day, all persons intending to leave Macao were required to submit a negative result of a COVID-19 Nucleic Acid PCR Test issued within 24 hours of departure, instead of the previous 7-day proof. Soon after, the border between Macao SAR (a Special Administrative Region of China guided by “one country, two systems” policy, see Ou & Sandel, 2021) and the Gongbei area of Zhuhai City (Chinese mainland) were put under a mandatory quarantine of 14 days. A female, border-commuting labor migrant, updated her WeChat *Moments* on August 4 by posting the following:


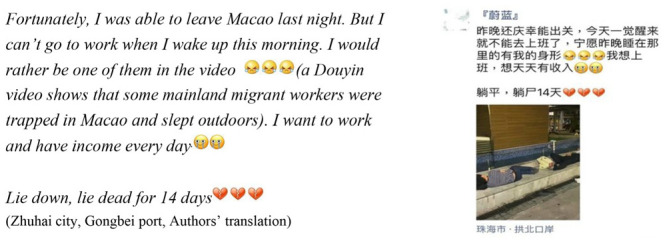


While the Coronavirus (COVID-19) pandemic has posed challenges to nations, governments, and global institutions, it is perhaps felt most acutely at the micro and individual levels. Individuals, especially those who must cross national or regional borders on a regular basis, are forced to navigate changing policies that have emerged during the pandemic, such as virus testing, quarantine, and restrictions on movement (e.g., Williams & Kayaoglu, 2020) . These changes of policies generate or strengthen the level of stress and risk for many, especially for subaltern groups whose lives pre-COVID were already marked by a high level of precarity (Ju et al., 2019; Ju & Sandel, 2020).

As reported in recent studies (Fisher & Ryan, 2021; United Nations [UN], 2020), COVID-19 has deepened pre-existing gender inequalities, and disproportionately impacted women and girls in such areas as the economy, health, security, social protection, and relational violence. Yet many women are on the frontlines, working in essential but low-paid jobs and dealing with the additional challenges of discrimination, inequality, insecure forms of labor, and racism (UN Women, 2020).

Coping with these challenges requires resilience. Broadly understood as the “ability of a system, community or society exposed to hazards to resist, absorb, accommodate to and recover from the effects of a hazard in a timely and efficient manner” (UN, 2009), governments, communities, and individuals need resilience in order to manage the stresses and risks of life during the COVID-19 pandemic. A tool many use for developing resistance is social media, as it can facilitate resilience building for those seeking medical information and social support during COVID-19 (Saud et al., 2020). Yet not all social media use is helpful. It can be a breeding ground for misinformation and discrimination (Pennycook et al., 2020; Yang et al., 2020). Therefore, it is important to understand social media use and its impacts during a time of crisis.

A growing body of research has explored social media use at different stages of disasters and emergencies (e.g., before, during, and after) by integrating information and communication technologies (ICTs) into crisis management (Alexander, 2013; Reuter & Kaufhold, 2018). Affected by the crises, individuals may alter their social media use patterns, and the impacts of which will also change over crises, including the COVID-19 pandemic (Kaya, 2020; Nguyen et al., 2020). Scholars have further argued that contextual factors, including features of online platforms, users’ socio-economic status and regional preferences, influence individuals’ social media use during a crisis (e.g., Goel & Gupta, 2020; Mano, 2020).

Focusing on a group of low-skilled migrant workers—female mainland cleaners in Macao, this study explored their uses of social media, and perceptions of its use during the COVID-19 pandemic. The findings will contribute to understanding how disadvantaged groups responded to the crisis, and develop gender-responsive and inclusive measures in coping with uncertainties and dynamic changes.

## Research Setting

The participants in this study are low-skilled labor migrants who left other provinces of rural China to seek higher-paid work in Macao, a Special Administrative Region (SAR) of China. Their place of employment and life circumstances are due to the labor policies and differential economic opportunities of Macao and surrounding regions. Yet higher wages are offset by the higher cost of living in Macao, when compared with the adjacent mainland city of Zhuhai. Therefore, to save on living expenses, most mainland workers migrate daily across the physical and political border that separates Macao (for work) from Zhuhai (for accommodations and most expenses).

As found in previous studies (Ju et al., 2019; Ju & Sandel, 2020), few participants have established friendships with Macao locals because of the time and long hours that mainland laborers spend at work (12 hours per day, 6 days a week) and crossing the border (two or more hours each day). Furthermore, they felt they were discriminated by local Macao residents due to their occupations, living habits that differed from local norms (e.g., queuing behavior, speaking loudly in public), and an identity as a Chinese “mainlander.” Macao was a place of work only, where many felt stigmatized and marginalized.

However, a benefit of the border crossing was that it enabled mainland migrant workers to access different social media platforms, since Macao was administrated under the “one country, two systems” policy. For instance, QQ Music and Youku work only in the mainland; Facebook, Twitter, and YouTube can be accessed only in Macao. However, WeChat, a multifunctional app used frequently by all participants, can be accessed on both sides of the border. This app’s widespread use, multiple functions, and accessibility provide mainland migrant workers with a common platform that they carry with them wherever they go, and afford affective support, access to information, and financial benefits (Ju et al., 2019).

Yet this patterned way of life was disrupted when the border was closed in February 2020 due to the first reported case of community spread of COVID-19 in Macao. In order to maintain their livelihoods, most mainland Chinese migrant workers chose to stay in Macao. They were stuck for about five months and then resumed border commuting when quarantine requirements were lifted. However, even after this, they were faced with uncertainty as policies could and did change in response to the virus (Suhardiman et al., 2021). As the WeChat *Moments* post at the beginning of this paper illustrates, these participants were at the risk of being homeless and jobless because of the crisis. To better understand how they resist and adapt to uncertainties and risks, we investigate their social media use.

## Literature Review

COVID-19 has posed unique challenges to females and deepened gender inequalities. Due to more time spent on homeschooling, household, and the gendered role of caregivers, women were found to suffer more loss in economic outcomes, exacerbated stress in mental health, reduced academic productivity, and increased exposure risk of being affected during the pandemic (Connor et al., 2020; Dang & Nguyen, 2021; Gabster et al., 2020). Among them, the female migrant workers, as the vulnerable population, were in worse condition. As a result of constrained mobility, women migrants experienced the loss of livelihood, withdrawal from migration flows and resulting debt, and the ensuing anxiety and fear made their life more stressful (Azeez et al., 2021; Song et al., 2021). The studies exploring how female migrants react and adjust themselves to the crisis are significant, and will accordingly provide gender-responsive support to enable both females and males to get through COVID-19 for building a diverse and inclusive society.

### The Ambivalence of Social Media in Resilience Building

The concept of resilience, based on the Latin word *resilio*, refers to the actions to “jump back” or “bounce back.” With its roots in ecological theory and complex adaptive systems, the notion of resilience has emerged as a framework for understanding the capacity and response to a stressor, which is more process-oriented than an outcome-oriented concept (Manyena, 2006; van Breda, 2018). The COVID-19 pandemic presents an opportunity to understand “resilience” as dynamic changes (“bounce forward”) rather than the static sameness as the original situations (“bounce back”).

Social resilience as an integrated approach to different societal levels (e.g., individual, family, and community) has been conceptualized and measured as a multi-dimensional construct. The development of social resilience requires structural properties like physical assets, economic resources, and personnel and cognitive attributes, including collective efficacy and a sense of community (Kwok et al., 2016). Furthermore, Saja et al. (2018) measured social resilience in the following five dimensions: social structure (e.g., social demography and mobility), social capital (e.g., social support), social mechanisms (e.g., community engagement), social equity (e.g., access to health), and social belief (e.g., cultural norms). Focusing on social structure, we argue that vulnerable groups will be more susceptible to hazards or stress because their socio-economic status and the restricted availability of support will constrain the extent and speed of their recovery from and adaptation to the crisis (Vinkers et al., 2020). They deserve more attention and help to achieve a resilient world in the long run.

With the proliferation of the Internet and mobile devices, the use of social media to build resilience in emergencies and disasters has drawn increasing attention, and its ambivalent roles have been identified (e.g., Alexander, 2013; Chu & Yang, 2020; Reuter et al., 2016). During the ongoing COVID-19 pandemic, the strength of social media has been adopted as real-time information access, information dissemination, and social support seeking across social groups. Take Cyprus as an example, they used social media as the primary medium to gain information, and the decision-makers adjusted the proposed legislation based on society’s reaction via social media (Kaya, 2020). Conversely, the unfavorable use of social media can lead to an infodemic, mental health problems, and the spread of negative sentiments like prejudice, discrimination, and hatred (Gao et al., 2020; Yang et al., 2020; Zarocostas, 2020). In responding to the ambivalent role of social media in the crisis, it is necessary to understand the social media use during the pandemic and device strategies to mitigate the impact brought by the social media panic (Depoux et al., 2020).

### Social Media Use and Women Migrant Workers: The Socio-Techno Approach

Social media use is closely related to a user’s material conditions (e.g., class, gender, nationality, and ethnicity). In existing literature, female migrant workers have mainly perceived social media as a means for empowerment (Cao & Li, 2018; Platt et al., 2016; Wallis, 2018). A socio-techno approach (Wallis, 2011b), highlighting the interplay between technology and socio-cultural practices, provides deeper insights into the social media used by female migrant workers in resilience.

Originating from the joint optimization of social (e.g., work culture, the occupational structure) and technical (e.g., automation, unit operations) systems (Emery, 1959), the Tavistock Institute’s social-technical systems emphasize the direct and correlative interactions of both systems in the outputs of the work organization (Trist, 1981). Therefore, any work system design must ensure an integrated form of social and technical systems. Later, this approach has been applied to manage technology design and organizational development by stressing the complex and dynamic interactions between humans, machines, and the environment (e.g., Baxter & Sommerville, 2011; Bostrom & Heinen, 1977), especially with the introduction of new technologies. Nowadays, the pervasiveness of smart mobile phones has deepened the penetration of social media into our everyday routine. van Dijck and Poell (2013) identified the social media logic underpinning the complex dynamic between social media platforms, users, mass media and social institutions.

Focusing on the interactions between social media and its users, domestication theory contributes to a further understanding of how users integrate technology into their everyday lives. Silverstone et al. (1992, p. 21) identified four parts in this fluid process: the initial acquisition of technology based on negation (appropriation), the location of technologies in the household (objectification), the use of technology into users’ routine and time structure (incorporation), and how users mobilize the technology as part of their identity and how to present themselves to others (conversion). By stressing the ongoing and endless domestication process, re-domestication characterizes a new role of technology in users’ lives when circumstances change, for distinguishing it from its first use (Haddon, 2003).

On the one hand, ICT use is constituted and/or restricted by the power structure wherein users live. The structural forces are intricate and could drive at the individual (e.g., constraints like social-economic status), social-cultural (e.g., hegemonic discourse), and political (e.g., state governance and the border regime) levels. As Indonesian domestic workers in Singapore have reported, their access and use of ICTs, including social media use, are subject to surveillance and regulation by their employers (Platt et al., 2016). On the other, ICT use may shape, preserve, weaken, or strengthen the existing power structure. In Wallis’ (2011a) study, though Chinese young female rural-urban migrant workers used mobile phones to connect with friends and family, and enhance their sense of community, their mobile phone use to some extent reinforced the constructed identity as migrants.

Apart from the dynamic between existing power structure and individuals’ ICT uses, users do have the power or agency (Giddens, 1984) to act against the structural constraints to meet their own needs based on the learned and stock knowledge. Taking WeChat *Group* as one of the examples, Pei and Chib (2021) have found that mobile phones empowered the Chinese female rural-urban migrant workers to negotiate agency for strategic responses like blocking family members to avoid patriarchal pressure, engaging with factory management, and forming alliances to challenge the uneven gender power relationships.

As Nguyen et al. (2020) have found, factors such as age, gender, Internet access, and skills were related to individuals’ changes in their digital media use during the pandemic. Therefore, investigating social media use in a resilient process should be associated with the co-constitution of users’ structural forces situated in a specific context. Though the lived experiences of females during the pandemic have been investigated in some studies, how social media is used by females, in particular low-skilled female migrant workers to cope with the crisis is underexplored. By taking the socio-techno approach, this study examines the following question:

What is the role of social media in the bordering crossing (in)activities during the COVID-19 for female mainland low-skilled laborers?

## Methods

We conducted a qualitative study to listen to the voices of female migrant workers. Macao has a labor shortage as a hybridized landscape boosted by the gambling industry, especially demanding for low-skilled occupations, such as construction, hotels, and restaurants (Ju & Sandel, 2020). Cleaners are the typical example of low-skilled workers engaged in so-called indecent jobs, and the majority of them are females. Therefore, a group of essential workers—female mainland laborers working in Macao as cleaners—were recruited for this study to exemplify how female migrant workers cope with the challenges posed by the pandemic. Ethics approval was obtained from the review board of the university with which the co-investigator (third author) was affiliated. The first author (a Chinese female) has known two participants since 2017 when conducting the previous work on their border-crossing life and kept in touch with them via WeChat. Their kind help enabled the recruitment of another 12 female migrant workers as participants, and recruitment ended when data saturation was reached. As shown in Table 1, the 14 participants aged from 20 to 50 years, and most were in their 30s and 40s. They had limited education, with most completing only junior high school, which since 1986 has been the minimum number of years of education in China (C. Tang et al., 2020). All were from the southern part of southern China and married or divorced with at least two children. The length of time working in Macao varied from less than 1 to 8 years.

**Table 1.** Participants’ Demographic Information.

Participants	Age	Sex	Education background	Marriage/children	Original place (city/province)	Length (/year) of Working in Macao	Job duties (cleaning)
1	20	Female	Junior high school	Married/3	Yunfu, Guangdong	<1	Teaching building
2	31	Female	Junior high school	Married/2	Jiangmen, Guangdong	1	Teaching building
3	35	Female	Junior high school	Married/2	Zhuhai, Guangdong	4	Sports stadium
4	35	Female	Technical secondary school	Married/2	Jiangmen, Guangdong	>2	Public toilet
5	38	Female	Junior high school	Married/2	Nanning, Guangxi	<1	Teaching building
6	39	Female	Primary school	Married/3	Zhanjiang, Guangdong	>2	Public toilet
7	43	Female	Junior high school	Married/2	Yangjiang, Guangdong	5	Teaching building
8	44	Female	Primary school	Married/2	Yunfu, Guangdong	>2	Public building
9	40-45	Female	Junior high school	Married/2	Yangjiang, Guangdong	>4	Public building
10	46	Female	Junior high school	Married/2	Changsha, Hunan	2	Public toilet
11	46	Female	Junior high school	Married/3	Jiujiang, Jiangxi	>1	Public toilet
12	47	Female	Junior high school	Married/3	Zhangjiajie, Hunan	8	Public toilet
13	50	Female	Junior high school	Divorced/3	Zhuzhou, Hunan	5	Student dormitory
14	51	Female	Junior high school	Married/2	Qingyuan, Guangdong	5	Hotels

Data were collected from semi-structured interviews. When restrictive border restrictions were lifted in July 2020, from August 2020 to November 2021 the first author conducted face-to-face interviews with participants at their worksites. As the semi-structured interview stresses the focused structure and reciprocity between the interviewer and participants (Kallio et al., 2016), participants were comfortable knowing what to share. The interviewer also was flexible in asking follow-up questions. Participants responded to the researcher-developed interview protocol of 13 questions that centered on four topics: challenges posed by the COVID-19 pandemic, use pattern of social media, impacts of social media, and changes in social media use. During interviews, participants were encouraged to narrate their stories relevant to social media use.

Fourteen interview recordings were transcribed for thematic analysis (Braun & Clarke, 2006) using NVivo 12. The coding process was conducted to categorize the empirical findings. Upon initial coding, the first author used inductive reasoning to examine the individual codes and the overarching themes through constant comparison (Glaser & Strauss, 1968). Analytical memoranda were kept throughout the coding process to delineate the linkages between the codes, note the decisions made on refining or adding new codes, and document the consistent interpretation of codes for forming the emerging themes. The analytical process produced five significant themes (see Table 2) on perceived social media use during the pandemic: (1) platforms of social media; (2) use during im/mobilities; (3) emotional health; (4) information access; and (5) risk management. Since the first two themes were general, they were further combined with the other three themes to highlight the role of social media in the following section. When quoting the participants’ words in Chinese, the researchers discussed and agreed upon the English version to avoid ambiguity and inaccuracy caused by cultural and language differences.

**Table 2.** Summary of Themes and Subthemes.

Themes	Subthemes
Social media platforms	*YouTube*: information from foreign countries; information censored in the mainland
	*Douyin*: killing time; funny video; positive energy; knowing the outside world; self-made video; cannot make money
	*WeChat*: access to COVID-19 information; video chat with family; online parenting; online counselling; online payment
	*Others* (e.g., Weibo; Xiaohongshu): news; entertainment
Use during im/mobilities	*Border closure*: daily use; several hours per evening; constant and addictive use
	*Border (re)opening*: frequency and length reduced
Emotion health	*Roles*
	Positive: depression and boredom alleviation; spousal connection; spiritual sustenance
	Negative: anxiety; disappointment; worry
Information access	*Multiple sources* (e.g., WeChat; Macao Daily; and Douyin)
	*False/fake information*
	Responding strategy: official information; no comments or no reposting when in doubt
	Challenge: difficult to distinguish; having no idea how to verify
Risk management	*Forms*
	Green code; daka; WeChat workgroup; phone call from the community
	*Attitudes*
	Positive: safety; support; indispensable
	Neutral: not afraid of being monitored; national policy; irrelevant to privacy
	Negative: inconvenient in border crossing

## Findings and Discussion

The participants experienced a stressful life mainly due to economic burdens and emotional stress/anxiety over border closures, which is consistent with the extant studies (Azeez et al., 2021; Liem et al., 2020; Song et al., 2021). As Macao-Zhuhai border commuters, they were exposed to more uncertainties and risks caused by the changing border policy and pandemic situations. Instead of passively constrained by predicament, they acted against the challenges by using social media on mobile phones which was perceived as an essential tool for them to develop resilience. Social media, however, was not a one-size-fits-all solution. A 43-year-old cleaner commented that her focus and concern were about making money that from hard work, rather than social media use. The more urgent issue for the participants was the border re-normalization which preconditioned their ability to earn higher income in Macao. Though social media alone could not ease the economic burden of female migrant workers, its empowering yet restricting role in resilience is demonstrated in the following three contrastive sections.

### Emotional Comfort Versus Anxiety Derived from Social Media Use

Participants used the functions of WeChat (*Audio/video Call*, *Group Chat*, and *Moments*) to gain familial and communal support during a time of waiting. Even though they said that they increased social media use, they still suffered from worry, insomnia, and helplessness. Aside from the high living cost in Macao, they had few/none local friends whom they could seek for help; they also were worried about being infected with the virus, especially when there was local spread of COVID-19 in Macao. Unlike their WeChat use on regular (i.e., pre-COVID, or when the border was reopened), participants actively sought emotional support and online psychological counselling via social media.

Late at night, I could use WeChat video chat to exchange feelings and maintain our spousal relationship. This interaction gives my empty heart a kind of sustenance (P9).

I was so bored when staying in Macao, so every day I touched here or there to search for the new functions of Alipay and WeChat. I used the health-related service embedded in WeChat Pay or Mini-Programs to find psychologists online, and it is free (P4).

To cope with anxiety and depression, YouTube was identified as a “tutor.” Participant 4 learned how to overcome negative feelings by taking some online courses (e.g., Harvard Open Course on Happiness), even though some courses were in English with Chinese subtitles that were not easy to read. (This participant’s experience may have been impacted by her background, as she had more years of formal schooling, and worked in Macao longer than the others.) In these ways, participants proactively negotiated agency and demonstrated strategic responses to a negative mood.

When most people in Macao were unable to eat in restaurants or socialize in public places because of COVID, participants spent their non-working time in small and crowded flats (e.g., 10 people living in a flat of one bedroom and one living room) rented in Macao. One of the few leisure-time activities available to them was browsing short videos on the app Douyin (TikTok outside of China) for viewing and posting short, 15- to 60-second videos (Kaye et al., 2021), with embedded tools (e.g., background music, stickers), that enable users to make professional-looking videos without special training. This helped them cope with boredom and negative emotions, and experience moments of joy and cheer.

Viewing videos via Douyin is a way to kill time. The funny videos will sometimes lift my mood. And many videos are about positive energy, such as how the volunteers and frontier workers fight against the COVID (P10).

Some participants learned how to post self-made videos via Douyin to express their voices. Most videos chronicling their life in Macao were used for self-entertainment. As Yan (2019) found, social media apps such as Douyin lower the barriers to uploading and sharing content, affording Chinese users with limited literacy a platform to share their stories.

However, participants also noticed the problems that they and others faced since their social media usage frequency and range increased. As Hou et al. (2020) reported, females experience more severe stress than males during the pandemic; females spending more than 60 minutes on pandemic-related information would increase the severity of anxiety. Some participants said they were “addicted” to Douyin use, which they described as constantly browsing the unending stream of videos; this was consistent with empirical findings of other studies that COVID-19 stress was positively correlated with addictive social media use (Zhao & Zhou, 2021). Moreover, the ubiquitous COVID-19 relevant information via social media aggravated negative feelings such as worry and disappointment. For instance, participants kept a close eye on the number of infected cases every day and desired to go back to Zhuhai. The newly reported cases in Macao and the quarantine policy often made them stressful. They were afraid of being blamed for carelessness at work, which may lead to more infected cases, and the hope of border reopening was disillusionment. However, the anxiety caused by social media use was relieved as soon as the border reopened, as they only worried about the new COVID-19 cases reported in Macao or Zhuhai.

### Social Media as Multiple Versus Unofficial Information Sources

Before COVID-19, mainland Chinese low-skilled labor migrants used WeChat mainly to seek information of personal interest and to acquire knowledge about Macao and immigration policies (Ju et al., 2019). During COVID-19, their information sources became more diversified. Since they were trapped in Macao, some started to follow Macao’s local news via social media and television. In addition to watching CCTV (China Central Television) news that was perceived to be authoritative and credible, the participants also got news and information from newly-followed local WeChat *Official Accounts* like 澳門日報 (Macao Daily), 在澳劳务人 (Mainland Migrant Workers in Macao), 珠海發佈 (Zhuhai Announcement), and 江門發佈 (Jiangmen Announcement). In addition, WeChat *Moments*, *Individual*/*Group Chat*, and Douyin were other platforms for them to get updated information from family, friends, or other users. When talking about the various information sources, participants emphasized the perceived differences between WeChat and Douyin.

It seems that WeChat could be mainly used to chat with people. But with Douyin, you can see many things that you don’t know, because people usually post everything on Douyin. You know that there is a lot of news to view, and there are many things to learn (P8).

Participants explained that Douyin is an ideal space for them to “walk out,” for example, to know the world outside, and learn how to get along and work with others. In contrast, WeChat seems more like “walk-in” place, where they have close contact with family and friends. Some also followed the news via YouTube and regarded it as a better channel to know the actual events occurring in foreign countries. Each social media platform’s affordance (Gibson, 1986) may link to further studies of social media use in the pandemic and migration.

COVID-19, to some extent, motivated and pushed the participants to expand their information network from local, regional, and even to global levels by accessing different social media platforms.

Anyway, it’s better for you to read more. “No harm in doing that, right?” If you don’t want to read it, just leave it there. If you want to know more, there is no harm in reading (P9).

During this time, social media facilitated participants’ exposure to multiple information sources, which empowered them with the knowledge to know the world during the pandemic. Also, the participants displayed their agency in deciding whether to learn or not.

Their agentic actions were, to some extent, constrained by limited literacy when the participants were bombarded with multiple sources of information, particularly when much false or misleading information was quickly and widely disseminated in digital environments. On the positive side, participants had developed their own rules to react. For example, they trusted the news released by official accounts or agencies, such as CCTV. If the news was reposted to other platforms like Douyin, the organization’s official logo was included and seen as credible. And they would not repost or forward unidentified information to others when they were required to do so.

However, they still lacked information literacy or acute awareness of the message from no reliable source. Most of the time, they just had a look and would not verify or evaluate its authenticity for two reasons. One was that the news had nothing to do with them, so checking was unnecessary. The other was related to their poor education. They had limited literacy and did not know how to verify sources.

I find it difficult to tell whether the information is true or fake. You can’t see it with your own eyes, can you? If it were misleading, just leave it there (P4).

Only take a look at the news when I am not sure whether it is true or not. And no judgment or comments on it (P6).

Noticeably, the participants had technical and digital competencies in social media, such as selecting accounts to follow and posting self-made Douyin videos. In terms of information without trustable sources, they did not repost or share it with others, especially during the outbreak of COVID-19. Yet, a lack of critical thinking and media skills limited the evaluation of the text itself. As Banerjee and Meena (2021) argued, social media is a double-edged sword in the pandemic, and vulnerable communities need to be protected from the effects of misinformation.

### Social Media in Risk Management Versus Surveillance

The role of social media as a health surveillance tool has been discussed in pandemic control (Calvo et al., 2020). Situated in border crossing, the real-time track of travel trajectory and the monitoring of population flow has become increasingly important. WeChat’s *Mini Program*, also accessible via *Official Account*, called 粤省事 (*Yue Sheng Shi*), is a requisite tool for different local governments in Guangdong Province to deliver information and provide public services (Z. Tang et al., 2021). One of the services offered during COVID-19 is the Guangdong health code. Participants obtained their personalized code (e.g., red code “mandatory quarantine,” yellow code “self-isolation,” or green code “free movement”) which not only tracks the result of nucleic acid testing, vaccination, and movement trajectory but facilitates users to convert and generate the green health code for eligibility in border crossing.

All participants accepted and supported the use of the health code in border crossing, although at first, its use seemed inconvenient.

There is no other solution. This is something you must comply with during the epidemic, right? It’s wise to be careful and protect yourself against the risks. If you were infected, the impact would be devastating. I think we should use the Guangdong health code (P7).

Aside from using social media to ensure safety at the government level, participants also talked about its use at the group and community levels. When they were trapped in Macao, their companies required them to report their locality every day via *Group Chat*, and emphasized the importance of staying at home and the COVID-19 prevention. After the Zhuhai-Macao border was opened, they needed to report their health conditions to the administrated community every day in the form of *daka* (打卡, to clock in). Participants were required to be involved with multiple reporting activities via WeChat. Though some surveillance studies scholars alerted the use of the global health crisis to “normalize” the surveillance measures by making them “necessary as insurance” to potential threats (French & Monahan, 2020, p. 6), none of the participants perceived WeChat as a surveillance tool violating their privacy but viewed it as a risk management instrument. They accepted the use of social media use tracking personal data and reported that doing so was to comply with the national policy. This finding is supported by a study by Liu (2021), which pointed out the Chinese public’s support for authority and domestic disease tracking.

Another type of social media monitoring was in participants’ workgroup via WeChat for working information sharing and management. They were required to report their work progress by uploading photos. And their mistakes were also posted, which sometimes made them feel embarrassed and lose face (see Pei & Chib, 2021). Yet workgroup surveillance was acceptable to participants: “This is a monitor but also my job. So, I am not afraid of being watched by others.”

### Implications of Findings

**Figure 1.**
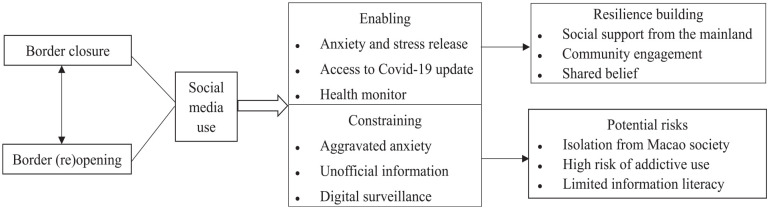
The role of social media perceived by female mainland low-skilled workers in Macao during the COVID-19.

To summarize, social media enabled and restricted female low-skilled labor migrants to build resilience during the pandemic (see Figure 1). The enabling role of social media among labor migrants during the pandemic is manifested: as a helpful tool in stress release, information access, and health monitoring. More specifically, women migrant workers used social media to connect well with family and friends, cope with anxiety and depression, share COVID-19 relevant information, and track their health conditions. Linking the findings with social resilience (Kwok et al., 2016; Saja et al., 2018), social media facilitates its users to gain social support, community engagement, and collective efficacy in resilience development.

Female migrant workers’ use of social media in resilience-building could be further explained by domestication theory focusing on the process of technology adoption and use. When exploring WeChat use, scholars stressed the users’ subjectivity, the specific context (concerning the dynamic between users and technology, and the issues like family, work, and social-cultural scenarios), and the ongoing process of technology use (Huang & Miao, 2020; Zhu & Miao, 2021). The multifaceted ways in which cultural or societal differences influence the dynamics of the domestication process pushed us to understand how social-cultural practices shape women migrants’ social media use in this study.

First, when staying in Macao as low-skilled migrant workers, participants actively used social media to gain the communal support that could be provided only by family and friends from the mainland. This further distanced them from Macao’s local society, and the longer they felt “trapped” in Macao, the greater their anxiety and negative emotions. Furthermore, “addictive” social media use increased stress severity, which in other studies has been found to be associated with lower socio-economic status (He et al., 2021).

Second, social media enabled participants to react proactively when receiving diversified and updated information relevant to the pandemic. But too many unofficial sources via social media reinforced the participants’ limitations in information literacy. This is consistent with the explanation made by Manca et al. (2021) that social media platforms can be socio-technical systems, and users’ information level is closely linked to individual and societal levels.

Third, participants readily accepted and advocated for social media as a risk management technology along with the expansive virus-tracking policy. To probe the reasons behind it, Liu and Zhao (2021) identified three factors: a paternalistic model of governance by caring and serving the people, a sense of cultural continuity in a collective vision, and communitarian values built upon individual responsibility, fidelity, and sacrifice. Public support for social media tracking system and its success in curbing the ongoing pandemic, to some extent, can maintain or strengthen the political and cultural beliefs shared among Chinese users.

Another concern worth noticing is that social media use varies with the users’ migratory circumstances during the pandemic (Nguyen et al., 2020). Compared with its role in pre-COVID days, WeChat continued to facilitate communal solidarity, an emotional mood of conviviality, and information access (Ju et al., 2019). Differentially, the featured use of social media was salient, manifested in coping with family issues such as marriage bonding. Moreover, they increased the use of social media to cope with anxiety and deficiency of COVID-19 information. They also generated their own rules for identifying fake or false information. After the Zhuhai-Macao border was opened, participants highlighted using social media in health monitoring instead of its frequent use to alleviate negative emotions due to their energy-draining border commuting.

The limitations of this study should also be noted. The primary one is that only the first author, who is most familiar with the participants, did the initial coding of the interview data, which other authors subsequently rechecked. Second, most participants in this study were mainly from Guangdong province due to its geographic proximity, and the reliance upon the first two participants’ personal connections. Participants from other regions in the mainland or who could not speak Cantonese may be recruited in future work to narrate their challenges when trapped in Macao. Third, all interviews were conducted in Mandarin, but some participants from Guangdong province were not very good at speaking it. The use of Cantonese in future research may have an impact on the willingness of Cantonese speakers to share deeper insights into their experiences.

## Conclusion

Restricted by low social-economic status and a nomadic lifestyle, female migrant workers were more susceptible to the changing context of COVID-19 (UN Women, 2020). Our study explores how female mainland low-skilled workers in Macao used social media to cope with the challenges posed by the pandemic. By taking a socio-techno approach, we have identified the enabling yet constraining role of social media in their responses to COVID-19, which is shaped by the users’ agentic actions, structural forces, and the ongoing pandemic. Findings from this study fill a gap in our understanding of social media use by female low-skilled workers as they adapt to the ongoing crisis, and further elaborate how technology dynamically interacts with users’ agency and structure (Giddens, 1984).

Overall, female mainland low-skilled labor migrants perceived social media as an essential and effective empowerment tool in their disrupted life. Multiple mobile phone-based social media apps (e.g., WeChat, Douyin, and YouTube) were used to suit their own needs and build resilience. For cross-border workers, the empowering role of social media in psychological counselling, community building, and COVID-19 information dissemination should be weaved into users’ migration journey. Furthermore, the participants’ roles as mother and wife, their educational background, and socio-economic status as low-skilled laborers in the Chinese context, articulate the featured use of social media in this study: online mothering and marriage bonding, emerging rules on unidentified information sources, and full compliance with using social media as a health surveillance tool to control the pandemic.

Furthermore, we should note the disempowerment of social media, which may reinforce the existing constraints and impede resilience due to the factors like women migrant workers’ high risk of addictive social media use and limited information literacy. In general, the users’ structural forces and agency situated in migration and pandemic shaped the paradoxical use of social media. Moreover, the ambivalent potential of social media and its incapacity to facilitate economic empowerment challenges the claims of techno-determinism, which fundamentally understands technology as the necessary or key factor for changes. Our findings emphasize the dynamic interplay of technology and power structures in a non-deterministic process. As a double-edged sword in the pandemic, we argue that social media do not make a radical socio-economic transformation for female low-skilled labor migrants, even though they actively used social media to withstand and adapt to risks. We call not only for a gender-inclusive approach to ICT empowerment, but recommend social protection from the government, more flexible policymaking (e.g., on immigration and employment systems), and meaningful use of social media to cope with risks and uncertainties. In doing so, the labor migrants could become more proactive and resilient to risk, rather than being passive and resistless, as shown in the opening description.
